# Estimate of COVID-19 Deaths, China, December 2022–February 2023

**DOI:** 10.3201/eid2910.230585

**Published:** 2023-10

**Authors:** Zhanwei Du, Yuchen Wang, Yuan Bai, Lin Wang, Benjamin John Cowling, Lauren Ancel Meyers

**Affiliations:** World Health Organization Collaborating Center for Infectious Disease Epidemiology and Control, School of Public Health, University of Hong Kong, Hong Kong, China (Z. Du, Y. Bai, B.J. Cowling);; Laboratory of Data Discovery for Health Limited, Hong Kong Science and Technology Park, Hong Kong (Z. Du, Y. Wang, Y. Bai, B.J. Cowling);; University of Cambridge, Cambridge, UK (Y. Wang);; University of Texas at Austin, Austin, Texas, USA (L.A. Meyers);; Santa Fe Institute, Santa Fe, New Mexico, USA (L.A. Meyers)

**Keywords:** SARS-CoV-2, mortality, China, COVID-19, importations, coronavirus disease, severe acute respiratory syndrome coronavirus 2, viruses, respiratory infections, zoonoses, vaccine-preventable diseases

## Abstract

China announced a slight easing of its zero-COVID rules on November 11, 2022, and then a major relaxation on December 7, 2022. We estimate that the ensuing wave of SARS-CoV-2 infections caused 1.41 million deaths in China during December 2022–February 2023, substantially higher than that reported through official channels.

For almost 3 years, China maintained a zero-COVID policy that effectively suppressed SARS-CoV-2 transmission. China began rolling back those rules on November 11, 2022, and ended most restrictions on December 7, 2022 (China Focus, 2023, https://english.news.cn/20221207/ca014c043bf24728b8dcbc0198565fdf/c.html), in response to the reduced severity of the Omicron variant or the growing socioeconomic and political costs of the restrictions. COVID-19 immediately surged; China reported nearly 82,000 COVID-19–related deaths during December 16, 2022–February 17, 2023 (*1*).

In December 2022, China disbanded its national COVID testing system and twice modified its criteria for classifying COVID-19–related deaths (*2*,*3*). The resulting uncertainties in reported occurrences and low official death counts have spurred speculation that official mortality reports from China substantially underestimate the full burden of the December 2022–January 2023 wave (*4*). In early December of 2022, the Chinese Center for Disease Control and Prevention (China CDC) launched a sentinel household surveillance program, tracking SARS-CoV-2 test positivity in 420,000 people in 22 provinces across China (*5*). We used those data to estimate a plausible range for the total number of COVID-19–related deaths during December 2022–January 2023. We classified a death as COVID-19–related if it occurred within 28 days of confirmed infection (*6*). 

## The Study

We estimated COVID-19–related deaths by using an individual-based simulation that incorporated daily test positivity reports from the China CDC sentinel household surveillance system during December 16, 2022–January 19, 2023. We also incorporated age-specific vaccination and boosting rates reported in China and published estimates of infection fatality rates, vaccine effectiveness, and rates of immunity waning. We built a stochastic model to generate COVID-19 death reports from infections occurring during December 8, 2022–January 19, 2023, in a population of 1 million persons whose ages were randomly assigned according to the national age distribution in China. Each simulation was based on the reported SARS-CoV-2 test positivity rate (*5*) to stochastically determine the number of persons who would have initially tested positive on that day. Those testing positive were assigned a vaccination history generated stochastically from the daily age-specific vaccination rates reported in China (*7*) and given a level of vaccine-acquired protection against death based on the date of their last dose and published estimates for vaccine effectiveness (*7*). The simulation used that value and the age-specific infection-fatality rate (Leung K, Leung GM, Wu J, unpub. data, https://www.medrxiv.org/content/10.1101/2022.12.14.22283460) to determine probabilistically whether the patiet died from COVID-19 (Appendix, https://wwwnc.cdc.gov/EID/article/29/10/23-0585-App1.pdf). Results were based on 1,000 model simulations. We conducted sensitivity analyses for assumed age-specific vaccine effectiveness (VE) against death, population size, and increase in infection-fatality rates as the healthcare system in China reached capacity.

The sentinel surveillance report from China CDC suggests that roughly 90% of China’s population were infected during the focal 35-day period (*5*). This large and rapid wave caused ≈1.41 (95% credibility interval [CrI] 1.14–1.73) million deaths across China; 0.80 (95% CrI 0.60–1.05) million of those deaths occurred among adults >80 years of age. Estimated COVID-19 mortality rates (per 1 million population) ranged from roughly 0 (95% CrI 0–17) among children <9 years of age to 22,400 (95% CrI 16,500–30,000) among adults >80 years of age (Figure; Appendix Tables 2, 3).

**Figure:**
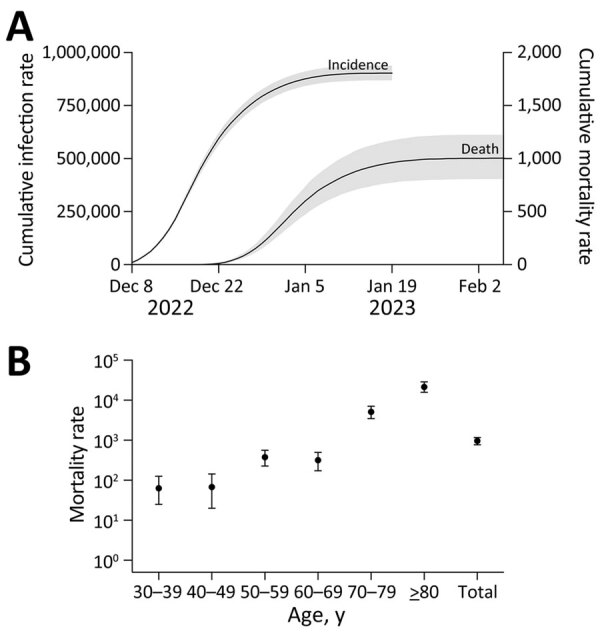
Estimated SARS-CoV-2 infection incidence in China during December 16, 2022–January 19, 2023, and resulting COVID-19 mortality rates. A) Estimated cumulative infection and mortality rates (per 1 million population) during December 8, 2022–February 7, 2023, based on test positivity data from the Chinese Center for Disease Control and Prevention sentinel community surveillance system, reported on January 26, 2023 (*5*). Gray shading indicates 95% credibility intervals derived from 1,000 stochastic simulations. B) Estimated age-specific COVID-19 mortality rates (deaths/1 million population, log scale), based on simulations that incorporate vaccine timing, coverage, effectiveness, and waning in each age group.

## Conclusions

COVID-19 deaths are related to a variety of health complications, including septic shock, multiorgan failure, respiratory failure, heart failure, and secondary infections (*8*). China’s official reports may underestimate the COVID-19 death toll by a factor of 17 (95% CrI 14–22). Our analyses suggest that, in barely a month, COVID-19 killed >1 million persons in China. The difference between China’s official mortality reports and our estimates may stem from delays in hospital reporting (*9*), omission of deaths happening outside of hospitals (*2*), gaps in China’s vital registration system (*4*), or intentional reclassification after the insurance industry in China largely stopped covering COVID-19 in December 2022 (South China Morning Post, December 17, 2022, https://www.scmp.com/news/china/science/article/3203695/chinas-covid-19-patients-face-insurance-battle-over-pandemic-related-payouts).

As our findings indicate, the relaxation of China’s zero-COVID policies in late 2022 precipitated an explosive wave of infections that caused an estimated 1,000 (95% CrI 843–1,230) deaths/1 million population. By comparison, during the large Omicron surges in early 2022, reported maximum 52-day mortality rates (deaths/1 million population) were 345 for the United States, 144 for the United Kingdom, and 1,166 for Hong Kong (*1*). Hong Kong’s high COVID-19 mortality rate may have resulted from its large proportion of older adults and relatively low vaccination rates in this vulnerable group; 26% of Hong Kong’s population is >60 years of age, and only 49% of that population had received >2 doses of a SARS-CoV-2 vaccine before March 2022 (*10*). By comparison, 90% of Australia’s population >60 years of age, which comprises 22% of the total population, were double-vaccinated by March 2022; the peak 52-day mortality rate in Australia was roughly 88% lower than that of Hong Kong (137 deaths/1 million population) (*10*). 

The unprecedented speed and severity of the wave in China is not surprising, given lack of infection-acquired immunity, moderate effectiveness of vaccines commonly administered in China, relatively low vaccine coverage in the oldest populations, and limited access to effective antiviral drugs. Mainland China had among the lowest estimated levels of excess mortality during the COVID-19 pandemic in 2020 and 2021 compared with 74 other countries worldwide (*11*), perhaps because of China’s dynamic zero-COVID strategy. The abrupt relaxation of zero-COVID rules without measures to protect high-risk populations likely led to the surge in hospitalizations and deaths we examined. As of June 21, 2023, the cumulative reported mortality rate in China is 85 deaths/1 million persons, considerably lower than rates for countries such as the United States (3,332/1 million persons), which sustained high levels of mortality before December 2022, and Japan (603/1 million persons), which experienced a substantial wave starting in December 2022, around the same time as China (E. Mathieu et al., 2020, https://ourworldindata.org/coronavirus). Our estimates suggest that China’s true death toll is closer to 1,014 deaths/1 million persons, roughly double that of Japan and 30% of that of the United States.

Our estimates are robust to moderate changes in the assumed age-specific vaccine efficacy and infection-fatality rates (Appendix Table 4). If the large surge in COVID-19 hospitalizations in late 2022 and early 2023 compromised patient care, we may have significantly underestimated the overall mortality rate. Assuming that COVID-19 mortality increased by a factor of 3.39 during China’s 3-day peak in reported test positivity (based on an estimate from a COVID-19 healthcare surge in Hong Kong in March 2022 [*12*]), our estimate of overall mortality increases to 2.11 (95% CrI 1.71–2.60) million.

Our findings rely on the validity of data from the China CDC’s sentinel household surveillance program, which might have some quality issues (e.g., double counting of persons who test multiple times). China CDC reports include graphs of daily positivity in this sample that enable rapid approximation of epidemic trends on a national scale (*5*). In addition, we assume that reported vaccinations were the only source of prior immunity and that all infections were by Omicron variants; surveillance data suggest that only 0.4% of specimens collected during this period were not Omicron (*5*). 

In summary, our study suggests that the official mortality reports from China substantially underestimate the full burden of the December 2022–January 2023 COVID-19 wave, raising concerns about the accuracy and transparency of China’s reporting system, as well as potential underestimation of reports from other countries that limit data collection and reporting. The decision to relax China’s zero-COVID policies without adequate measures to protect high-risk populations had severe consequences. Other countries prioritized vaccines for older age groups and other vulnerable populations (*13*), and many studies have indicated that targeting medical countermeasures and protective measures toward groups with high infection-fatality rates can be life and cost saving (*14*,*15*). We expect that the true toll of COVID-19 in China will become clearer as additional epidemiologic data become available.

Appendix Additional information for estimate of COVID-19 deaths, China, December 2022–February 2023.
