# Supplementary material for: Estimate of COVID-19 Deaths, China, December 2022–February 2023
Source: Emerg Infect Dis. 2023 Oct;29(10):2121–4. doi: 10.3201/eid2910.230585 (PMC10521589; doi:10.3201/eid2910.230585)
Supplement: Appendix — Additional information for estimate of COVID-19 deaths, China, December 2022–February 2023. [file 23-0585-Techapp-s1.pdf]

Article DOI: http://doi.org/10.3201/eid2910.230585

EID cannot ensure accessibility for supplementary materials supplied by authors. Readers who have difficulty accessing supplementary content should contact the authors for assistance.

Estimate of COVID-19 Deaths, China, December 2022–February 2023

Appendix

Estimating Omicron COVID-19 mortality in China based on sentinel surveillance data from December 16, 2022, to January 19, 2023

We ran 1000 simulations each with one million individuals assigned ages according to the national age distribution in China. Each simulation produces an estimated number of COVID-19 deaths resulting from infections occurring between December 8, 2022 and January 19, 2023, as described below. We report the 2.5th percentile (lower CrI bound), median, and 97.5th percentile (upper CrI bound) values across the 1000 simulations.

The full parameter specification is given in Appendix Table 1. In each simulation, we do the following:

- For each age group a, select a random IFR (IFR_a) from the estimated distributions given in Table S1 and assign each individual their age-specific IFR. (For each age group, draw from triangle distributions with lower bound, mode, and upper bound equal to the corresponding lower CI, mean, and upper CI, respectively.)
- For each day between December 16, 2022, and January 19, 2023, we use the reported SARS-CoV-2 test positivity from the China CDC sentinel surveillance system *(1)* to determine a random number of people in our simulated population of one million who would have first tested positive on that day. Specifically, for each day, we estimate the confidence interval for the reported test positivity *(1)* assuming a sample size of 2,500 (i.e., the reported minimum number of individuals in each community participating in the sentinel surveillance system). We then determine the number of newly positive

individuals by drawing a random deviate from the normally distributed sampling distribution for the test positivity statistic and multiplying that number by one million.

For each of those individuals, we determine their date of infection assuming that the earliest possible date was December 8, 2022 (restrictions ended on December 7, 2022 *(2)*), as follows:

- ○ Track time in terms of the number of days after December 7 and use t_{pos} to denote the number of days between December 7 and the day on which the individual first tested positive.
- ○ Assume that they tested negative in the prior sampling period. For example, an individual first testing positive on December 24 ($t_{\text{pos}} = 17$) presumably tested negative during the December 20–22 and the December 16–19 sampling periods. Randomly assign dates in each of those periods for their negative tests. We use T_{neg} to denote the vector of negative test dates, where dates are again represented by the number of days since December 7.
- ○ Let $P_{\text{pos}}(t_{\text{test}} - t_{\text{inf}})$ denote the probability of testing positive on day t_{test} given infection on day t_{inf} (Table S1). Determine the probability of having been infected on day t_{inf}, given negative tests on T_{neg} and a positive test on t_{pos} as given by

$$P(t_{\text{inf}}|T_{\text{neg}}, t_{\text{pos}}) = \frac{P(T_{\text{neg}}, t_{\text{pos}}|t_{\text{inf}})(P(t_{\text{inf}}))}{P(T_{\text{neg}}, t_{\text{pos}})}$$

where

$$P(T_{\text{neg}}, t_{\text{pos}}|t_{\text{inf}}) = P_{\text{pos}}(t_{\text{pos}} - t_{\text{inf}})\Pi_{u \in T_{\text{neg}}}(1 - P_{\text{pos}}(u - t_{\text{inf}}))$$

$$P(t_{\text{inf}}) = \frac{1}{t_{\text{pos}}}$$

$$P(T_{\text{neg}}, t_{\text{pos}}) = \frac{1}{t_{\text{pos}}} \sum_{t=1}^{t_{\text{pos}}} (P(T_{\text{neg}}, t_{\text{pos}}|t))$$

where $P(t_{\text{inf}})$ denotes the base probability that an individual was infected on day t_{inf} in the absence of information about their testing history and is assumed to be uniformly distributed over all days between December 7 and the day they tested positive; $P(T_{neg}, t_{pos})$ denotes the

probability of having negative tests on T_{neg} followed by a positive test on t_{pos} and is obtained by averaging the probability of a case experiencing both T_{neg} and t_{pos} given that they were infected on day *t* ($P(T_{neg}, t_{pos}|t)$) over all days *t* in between December 7 and the day they tested positive.

- Use this probability distribution to randomly assign an infection date.

- For each positive individual, determine their vaccination history according to reported daily age-specific vaccination rates in China, as follows *(3)*:
  - Randomly select the date of the first dose (t_1) based on the estimated first-dose rate, $C^1{}_a(t)$.
    - For children aged 3 to 11, first doses started on November 1, 2021.
    - For children aged 12 to 17, first doses started on August 1, 2021.
    - For adults aged 18 to 59, first doses started on December 1, 2020.
    - For adults aged over 60, first doses started on April 1, 2021.
  - Randomly select the date of the second dose (t_2) based on the estimated second-dose rate, $C^2{}_a(t)$, beginning 3 weeks after their first dose *(4)*.
  - Randomly select the date of the booster dose (t_3) for adults aged over 18 according to the estimated booster rate, $C^3{}_a(t)$, starting at the CDC-recommended time waiting period after their second dose (i.e., 6 months before December 4, 2022, and 3 months after December 5, 2022*(5)*).
- For each positive individual, determine their level of vaccine-acquire protection against death based on the date of their last dose and published estimates for vaccine effectiveness (VE). Assume that vaccine-acquired protection begins 2 weeks after each dose has been administered and that protection wanes stepwise 6 months following each dose *(3)*.
- For each positive individual, probabilistically determine whether they died from COVID based on their IFR and vaccine-acquired protection. If so, determine the day of death as follows:
  - Randomly select the date of symptom onset based on the estimated distribution of incubation periods (D_{inc}).

  - Randomly select the date of death based on the estimated distribution of days from symptom onset to death (D_{death}) *(6)*.
- Scale total deaths from a simulated population of 1M to the entire population of China by age group.

References

1. Chinese Center for Disease Control and Prevention. COVID- 19 Clinical and surveillance data–Dec 9, 2022 to Jan 23, 2023, China. chinacdc. 2023 [cited 2023 Jan 28]. https://en.chinacdc.cn/news/latest/202301/t20230126_263523.html
2. China Focus. COVID-19 response further optimized with 10 new measures. [cited 2023 Feb 4]. https://english.news.cn/20221207/ca014c043bf24728b8dcbc0198565fdf/c.html
3. Cai J, Deng X, Yang J, Sun K, Liu H, Chen Z, et al. Modeling transmission of SARS-CoV-2 Omicron in China. Nat Med. 2022;28:1468–75. PubMed https://doi.org/10.1038/s41591-022-01855-7
4. Covid-Vaccine Technical Working Group. Technical Vaccination Recommendations for COVID-19 Vaccines in China (First Edition). China CDC Wkly. 2021 May 21;3(21):459–61.
5. China Joint Prevention and Control Mechanism of the State Council. Strengthen the work plan for the COVID-19 vaccination of the elderly, 2023 [cited 2023 Jun 5]. https://www.samr.gov.cn/ltb/tzgg/art/2022/art_3216bd0086c74d70a55cc25891ec1b05.html
6. HKUMed updates on modelling the fifth wave of COVID-19 in Hong Kong (Update #8 as of March 14, 2022). [cited 2022 Apr 25]. https://sph.hku.hk/en/News-And-Events/Press-Releases/2022/TBC
7. National Bureau of Statistics of China. China Statistical Yearbook 2021. stats.gov.cn. [cited 2023 Jan 28]. http://www.stats.gov.cn/tjsj/ndsj/2021/indexch.htm
8. Mefsin Y, Chen D, Bond HS, Lin Y, Cheung JK, Wong JY, et al. Epidemiology of infections with SARS-CoV-2 Omicron BA.2 variant in Hong Kong, January-March 2022. https://doi.org/10.1101/2022.04.07.22273595
9. Chu VT, Schwartz NG, Donnelly MAP, Chuey MR, Soto R, Yousaf AR, et al.; COVID-19 Household Transmission Team. Comparison of Home Antigen Testing With RT-PCR and Viral Culture During the Course of SARS-CoV-2 Infection. JAMA Intern Med. 2022;182:701–9. PubMed https://doi.org/10.1001/jamainternmed.2022.1827

10. National Health Commission. Transcript of Press Conference of the State Council's Joint Prevention and Control Mechanism for the Novel Coronavirus Pneumonia Outbreak (November 29, 2022). 2022 [cited 2023 Jan 30]. http://www.nhc.gov.cn/xcs/s3574/202211/6fedb556a9324cd3b5b986446ee7ca34.shtml

11. Leung K, Leung GM, Wu J. Modelling the adjustment of COVID-19 response and exit from dynamic zero-COVID in China. medRxiv. 2022 [cited 2022 Dec 14]. https://www.medrxiv.org/content/10.1101/2022.12.14.22283460.abstract https://doi.org/10.1101/2022.12.14.22283460

12. COVID-19 Forecasting Team. Variation in the COVID-19 infection-fatality ratio by age, time, and geography during the pre-vaccine era: a systematic analysis. Lancet. 2022;399:1469–88. PubMed https://doi.org/10.1016/S0140-6736(21)02867-1

13. Wong JY, Cheung JK, Lin Y, Bond HS, Lau EHY, Ip DKM, et al. Intrinsic and effective severity of COVID-19 cases infected with the ancestral strain and Omicron BA.2 variant in Hong Kong. medRxiv. 2023. [cited 2023 Feb 13]. https://www.medrxiv.org/content/10.1101/2023.02.13.23285848v1.full https://doi.org/10.1101/2023.02.13.23285848

14. McMenamin ME, Nealon J, Lin Y, Wong JY, Cheung JK, Lau EHY, et al. Vaccine effectiveness of one, two, and three doses of BNT162b2 and CoronaVac against COVID-19 in Hong Kong: a population-based observational study. Lancet Infect Dis. 2022;22:1435–43. PubMed https://doi.org/10.1016/S1473-3099(22)00345-0

Appendix Table 1. Model parameters and data sources.

Symbol	Description	Values	Sources
N_a	Age-specific population size in China	Age 0–9: 168127944<br>Age 10–19: 157940134<br>Age 20–29: 166789007<br>Age 30–39: 223158122<br>Age 40–49: 207180217<br>Age 50–59: 222565082<br>Age 60–69: 147388498<br>Age 70–79: 80828885<br>Age ≥80: 35800835	China Statistical Yearbook 2021 *(7)*
D_{inc}	Incubation period	Triangular (4.1, 4.58, 5.08) days	Ref. *(8)*
D_{death}	Days from symptom onset to death	Lognormal (10.5, 0.043) days	Ref. *(6)*
$P_{\text{test}}(t)$	Probability of testing positive *t* days after initial infection	Test positivity *t* days after infection	Derived combining values given in Figure 1 in Ref. *(9)* for the daily PCR-RT positive rate post symptom onset and the distribution of incubation periods (D_{inc})
$I_{\text{tot}}(t)$	Proportion of the population newly infected at time *t*	Daily positive rate between December 16, 2022 to January 19, 2023	Extracted from Figure 1–5 in Ref. *(1)*.
$C^i_a(t)$	Age-specific vaccine coverage of the i-th dose (first, second, and booster) from December 2020 to September 2022 in China		We assume the cumulative vaccination rates of the first, second, and booster doses before March 1, 2022 follows the published values in Ref. *(3)*.<br>For adults <60 y, cumulative vaccination coverage hardly changes between March and December of 2022. For adults ≥60 y, cumulative vaccination rates for first, second, and booster doses are reported as 90.68%, 86.42%, and 68.8%, respectively, as of November 28, 2022 *(10)*, and 96%, 96%, and 92% as of January 20, 2023 *(1)*.<br>We assume a constant daily rate of vaccine administration during this period.
$VE(t)$	Vaccine effectiveness (VE) against mortality for an individual with most recent dose administered at time *t*, as of December 2022 in China	First dose: after two weeks 53.0%; after six months 53.0%<br>Second dose: after two weeks 66.3%; after six months 59.7%<br>Booster dose: after two weeks 79.2%; after six months 76.3%	Ref. *(3)*
IFR_a	Age-specific infection-fatality (IFR) without vaccination or antiviral treatment	Age 0–9: 0.0005% (95% CI: 0.0004%, 0.0008%)<br>Age 10–19: 0.0005% (95% CI: 0.0003%, 0.0008%)<br>Age 20–29: 0.0005% (95% CI: 0.0004%, 0.0008%)<br>Age 30–39: 0.023% (95% CI: 0.016%, 0.034%)<br>Age 40–49: 0.023% (95% CI: 0.016%, 0.036%)<br>Age 50–59: 0.126% (95% CI: 0.088%, 0.196%)<br>Age 60–69: 0.126% (95% CI: 0.087%, 0.198%)<br>Age 70–79: 2.00% (95% CI: 1.38%, 3.15%)<br>Age ≥80: 8.70% (95% CI: 6.12%, 13.01%)	Mean values are based on estimates in Ref. *(11)*.<br>95% confidence intervals are derived from Ref. *(12)* which estimates age-specific IFR's at 10 y intervals (ages 5, 15, 25 …) between April 15, 2020 and January 1, 2021, before broad vaccination and the emergence of the Delta and Omicron variants. Specifically, we use the ratios of the lower and upper CI's to the mean in Ref. *(12)* to scale the estimates in Ref. *(11)*. For example, consider the 70–79 age group. The estimate of 4.84% (95% CI: 3.33%, 7.63%) given in Ref. *(12)* for 75 y olds yields ratios of 0.69 to 1.58. We use these values to scale the mean for 70–79 y olds in Ref. *(11)* to obtain 2.00% (95% CI: 1.38%, 3.15%).

Appendix Table 2. Estimated age-specific COVID mortality in China due to the December 2022–January 2023 wave. We estimate the total numbers of deaths occurring in each age group.

Age group, y	Total deaths, median [95% CrI]
0–9	0 [0 - 2860]
10–19	0 [0 - 2820]
20–29	0 [0 - 2820]
30–39	15500 [5640 - 29600]
40–49	14100 [4230 - 29600]
50–59	87400 [53600 - 130000]
60–69	49300 [26800 - 76100]
70–79	431000 [292000 - 595000]
≥80	802000 [592000 - 1070000]
Total	1410000 [1140000 - 1730000]

Appendix Table 3. Estimated age-specific COVID deaths per million people in China due to the December 2022–January 2023 wave. We estimate the overall death rate for each age group.

Age group, y	Total rate, median [95% CrI]
0–9	0 [0 - 17]
10–19	0 [0 - 18]
20–29	0 [0 - 17]
30–39	69 [25 - 133]
40–49	68 [20 - 143]
50–59	393 [241 - 583]
60–69	335 [182 - 517]
70–79	5340 [3610 - 7360]
≥80	22400 [16500 - 30000]
Total	1000 [807 - 1220]

Appendix Table 4. Results of Sensitivity Analyses. Values are the estimated median [95% CrI] in million deaths across China between December 2022 and February 2023 based on 1000 stochastic simulations. Each scenario (S1-S6) changes one of the base assumptions, as indicated in the second column.

		Estimated deaths (millions)	
Scenarios	Description	>80 years age group	Total
Base	VE_a against mortality and IFR_a as specified in Table 1; Population of 1 million	0.80 [0.59 - 1.07]	1.41 [1.14 - 1.73]
S1	Ineffective vaccines: VE_a = 0% for all primary and booster doses	2.93 [2.16 - 3.91]	5.11 [4.15 - 6.28]
S2	Durable vaccines: VE_a does not decline after six months	0.76 [0.55 - 1.01]	1.32 [1.06 - 1.62]
S3	Surge-related increase in mortality rate: IFR_a increases 3.39-fold December 20–22 ⁺.	1.21 [0.89 - 1.62]	2.11 [1.71 - 2.60]
S4	Population of 2 million	0.82 [0.61 - 1.09]	1.43 [1.16 - 1.76]
S5	Population of 500 thousand	0.82 [0.59 - 1.09]	1.43 [1.13 - 1.76]
S6	Alternative age-specific VE_a*	0.90 [0.66 - 1.21]	1.56 [1.28 - 1.93]

⁺ The weekly hospitalization fatality risk was estimated to be 3.39 times higher at the March 2022 COVID-19 peak in Hong Kong relative to estimates from the end of October 2022, after the wave had subsided *(13)*. We assume that the IFR_a's increase by this amount during the three-day peak in the average daily positive rate reported by China *(1)*.

* Our Base scenario assumes the vaccines afford the same level of protection against mortality across all age groups. (i.e., Reduction in mortality risk following the **first dose**: after two weeks 53.0%; after six months 53.0%; **second dose**: after two weeks 66.3%; after six months 59.7%; **booster dose**: after two weeks 79.2%; after six months 76.3%). Scenario S6 assumes variable levels across age groups, derived from the following estimates provided in Ref. *(14)*: VE_a against mortality for the [60–69y, 70–79y, 80+y] age groups relative to that for 20–59y is: [84%, 58%, 57%] after the first dose, [90%, 82%, 68%] after the second dose, and [100%, 0.98%, 0.98%] after the third dose. We scale the Base VE's by these estimates to obtain the VE_a assumed in S6 for the [0–59y, 60–69y, 70–79y, 80+y] groups:

- First dose: after two weeks 53.0%·[100%, 84%, 58%, 57%]; after six months 53.0%·[100%, 84%, 58%, 57%]
- Second dose: after two weeks 66.3%·[100%, 90%, 82%, 68%]; after six months 59.7%·[100%, 90%, 82%, 68%]
- Booster dose: after two weeks 79.2% · [100%,100%, 0.98%, 0.98%]; after six months 76.3%· [100%, 100%, 0.98%, 0.98%]
